# A Deep Learning Approach for Automatic Seizure Detection in Children With Epilepsy

**DOI:** 10.3389/fncom.2021.650050

**Published:** 2021-04-08

**Authors:** Ahmed Abdelhameed, Magdy Bayoumi

**Affiliations:** Department of Electrical and Computer Engineering, University of Louisiana at Lafayette, Lafayette, LA, United States

**Keywords:** deep learning, epileptic seizure detection, EEG, autoencoders, classification, convolutional neural network (CNN), bidirectional long short term memory (Bi LSTM)

## Abstract

Over the last few decades, electroencephalogram (EEG) has become one of the most vital tools used by physicians to diagnose several neurological disorders of the human brain and, in particular, to detect seizures. Because of its peculiar nature, the consequent impact of epileptic seizures on the quality of life of patients made the precise diagnosis of epilepsy extremely essential. Therefore, this article proposes a novel deep-learning approach for detecting seizures in pediatric patients based on the classification of raw multichannel EEG signal recordings that are minimally pre-processed. The new approach takes advantage of the automatic feature learning capabilities of a two-dimensional deep convolution autoencoder (2D-DCAE) linked to a neural network-based classifier to form a unified system that is trained in a supervised way to achieve the best classification accuracy between the ictal and interictal brain state signals. For testing and evaluating our approach, two models were designed and assessed using three different EEG data segment lengths and a 10-fold cross-validation scheme. Based on five evaluation metrics, the best performing model was a supervised deep convolutional autoencoder (SDCAE) model that uses a bidirectional long short-term memory (Bi-LSTM) – based classifier, and EEG segment length of 4 s. Using the public dataset collected from the Children’s Hospital Boston (CHB) and the Massachusetts Institute of Technology (MIT), this model has obtained 98.79 ± 0.53% accuracy, 98.72 ± 0.77% sensitivity, 98.86 ± 0.53% specificity, 98.86 ± 0.53% precision, and an F1-score of 98.79 ± 0.53%, respectively. Based on these results, our new approach was able to present one of the most effective seizure detection methods compared to other existing state-of-the-art methods applied to the same dataset.

## Introduction

Epilepsy is inevitably recognized to be one of the most critical and persistent neurological disorders affecting the human brain. It has spread to more than 50 million patients of various ages worldwide (World Health Organization, 2020) with approximately 450,000 patients under the age of 17 in the United States out of nearly 3 million American patients diagnosed with this disease (Epilepsy in Children, 2020). Epilepsy can be characterized apparently by its recurrent unprovoked seizures. A seizure is a period of anomalous, synchronous innervation of a population of neurons that may last from seconds to a few minutes. Epileptic seizures are ephemeral instances of partial or complete abnormal unintentional movements of the body that may also be combined with a loss of consciousness. While epileptic seizures rarely occur in each patient, their ensuing effects on the patients’ emotions, social interactions, and physical communications make diagnosis and treatment of epileptic seizures of ultimate significance.

Electroencephalograms (EEGs; Schomer and Lopez da Silva, 2018) which have been around for a long time, are commonly used among neurologists to diagnose several brain disorders and in particular, epilepsy attributable to workable reasons, such as its availability, effortlessness, and low cost. EEG operates by positioning several electrodes along the surface of the human scalp and then recording and measuring the voltage oscillations emanating from the ion current flowing through the brain. These voltage oscillations, which correspond to the neuronal activity of the brain, are then transformed into multiple time series called signals. EEG is a very powerful non-invasive diagnostic tool since we can use it precisely to capture and denote epileptic signals that are characterized by spikes, sharp waves, or spike-and-wave complexities. As a result, EEG signals have been the most widely used in the clinical examination of various epileptic brain states, for both the detection and prediction of epileptic seizures.

By interpreting the recorded EEG signals visually, neurologists can substantially distinguish between epileptic brain activities during a seizure (ictal) state and normal brain activities between seizures (interictal) state. Over the last two decades, however, an abundance of automated EEG-based epilepsy diagnostic studies has been established. This was motivated by the exhausting and time-consuming nature of the human visual evaluation process that depends mainly on the doctors’ expertise. Besides that, the need for objective, rapid, and effective systems for the processing of vast amounts of EEG recordings has become unavoidable to be able to diminish the possibility of misinterpretations. The availability of such systems would greatly enhance the quality of life of epileptic patients.

Following the acquisition and pre-processing of EEG raw signals, most of the automated seizure detection techniques consist of two key successive stages. The first stage concerns the extraction and selection of certain features of the EEG signals. In the second step, a classification system is then built and trained to utilize these extracted features for the detection of epileptic activities. The feature extraction step has a direct effect on the precision and sophistication of the developed automatic seizure detection technique. Due to the non-stationary property of the EEG signals, the feature extraction stage typically involves considerable work and significant domain-knowledge to study and analyze the signals either in the time domain, the frequency domain, or in the time-frequency domain (Acharya et al., 2013). Predicated on this research, it has become the mission of the system designer to devise the extraction of the best-representing features that can precisely discriminate between the epileptic brain states from the EEG signals of different subjects.

In the literature, several EEG signal features extracted by various methods have been proposed for seizure detection. For example (Song et al., 2012), used approximate entropy and sample entropy as EEG features, and integrated them with an extreme learning machine (ELM) for the automated detection of epileptic seizures. Chen et al. (2017) used non-subsampled wavelet–Fourier features for seizure detection. Wang et al. (2018) proposed an algorithm that combines wavelet decomposition and the directed transfer function (DTF) for feature extraction. Raghu et al. (2019) proposed using matrix determinant as a feature for the analysis of epileptic EEG signals. Certainly, even with the achievement of great results, it is not inherently guaranteed that the features derived through the intricate, and error-prone manual feature extraction methodology would yield the maximum possible classification accuracy. As such, it would be very fitting to work out how to build substantial systems that can automatically learn the best representative features from minimally preprocessed EEG signals while at the same time realize optimum classification performance.

The recent advances in machine learning science and particularly the deep learning techniques breakthroughs have shown its superiority for automatically learning very robust features that outperformed the human-engineered features in many fields such as speech recognition, natural language processing, and computer vision as well as medical diagnosis (Wang et al., 2020). Multiple seizure detection systems that used artificial neural networks (ANNs) as classifiers, after traditional feature extraction, were reported in previous work. For instance (Orhan et al., 2011), used multilayer perceptron (MLP) for classification after using discrete wavelet transform (DWT) and K-means algorithm for feature extraction. Samiee et al. (2015) also used MLP as a classifier after using discrete short-time Fourier transform (DSTFT) for feature extraction. In Jaiswal and Banka (2017), ANNs were evaluated for classification after using the local neighbor descriptive pattern (LNDP) and one-dimensional local gradient pattern (1D-LGP) techniques for feature extraction. Yavuz et al. (2018) performed cepstral analysis utilizing generalized regression neural network for EEG signals classification. On the other hand, convolutional neural networks (CNNs) were adopted for both automatic feature learning and classification. For example (Acharya et al., 2018), proposed a deep CNN consisting of 13 layers for automatic seizure detection. For the same purpose (Abdelhameed et al., 2018a), designed a system that combined a one-dimensional CNN with a bidirectional long short-term memory (Bi-LSTM) recurrent neural network. Ke et al. (2018); Zhou et al. (2018), and Hossain et al. (2019) also used CNN for feature extraction and classification. In Hu et al. (2019), CNN and support vector machine (SVM) were incorporated together for feature extraction and classification of EEG signals.

As reported, most of the deep learning algorithms that involved automatic feature learning have targeted single-channel epileptic EEG signals. It is therefore still important to research more data-driven algorithms that can handle more complex multichannel epileptic EEG signals.

In general, supervised learning is the most widely used technique for classifying EEG signals among all other machine learning techniques. Several researchers have recently experimented with semi-supervised deep learning strategies in which an autoencoder (AE) neural network can benefit from training using both unlabeled and labeled data to improve the efficacy of the classification process (Gogna et al., 2017; Yuan et al., 2017; Abdelhameed and Bayoumi, 2018, 2019; She et al., 2018). Two approaches of using AEs have been used in the literature. The first one is the stacked AEs approach, where each layer of a neural network consisting of multiple hidden layers is trained individually using an AE in an unsupervised way. After that, all trained layers are stacked together and a softmax layer is attached to form a stacked network that is finally trained in a supervised fashion. The second approach uses deep AEs to pre-train all layers of the neural network simultaneously instead of that greedy layer-wise training. This latter approach still suffers from one particular drawback which is the necessity to train the semi-supervised deep learning model twice. One training episode is conducted in an unsupervised way using unlabeled training data that enables the AE to learn good initial parameters (weights). In the second episode, the first half of the pre-trained AE (the encoder network) attached to a selected classifier is trained as a new system in a supervised manner using labeled data to perform the final classification task.

Therefore, in this work, to address the limitation of the classification schemes alluded to above, a novel deep learning-based system that uses a two-dimensional supervised deep convolutional autoencoder (2D-SDCAE) is proposed for the detection of epileptic seizures in multichannel EEG signals recordings. The innovative approach in the proposed system is that the AE is trained only once in a supervised way to perform two tasks at the same time. The first task is to automatically learn the best features from the EEG signals and to summarize them in a succinct, low-dimensional, latent space representation while performing the classification task efficiently. The method of consolidating the simultaneous learning to perform both tasks in a single model, which is trained only once in a supervised way, has proven to have a good impact on improving the learning capabilities of the model and thus achieving better classification accuracy.

In addition to operating directly on raw EEG signal data, there are several advantages to our approach. First of all, the SDCAE is faster compared to conventional semi-supervised classification systems since it is trained only once. Second, to minimize the total number of network parameters, the proposed SDCAE uses convolutional layers for learning features instead of fully connected layers that are commonly used in regular MLP-based AEs. Third, the proposed system can be used in signal compression schemes as the original high-dimensional signals can be perfectly reconstructed from the low-dimensional latent representation using the second half of the AE (the decoder network). Finally, the training of AEs in a supervised way is more effective in learning more structured latent representation, making it very feasible to deploy very simple classifiers and still have very high-precision seizure detection systems. It is also worth noting that performance and hardware resource-saving have been taken into account to make the proposed system more suitable for real-time use and potential hardware implementation and deployment.

Two SDCAE models are designed to test our novel approach, and their performance for seizure detection in children is evaluated. Both models are used to classify EEG data segments to distinguish between ictal and interictal brain states. The first model is a two-dimensional deep convolution autoencoder (2D-DCAE) in which the convolutional layers of the encoder network are attached to a simple MLP network consisting of two fully connected hidden layers and one output layer for classification. The second system is also a 2D-DCAE but in this system, the convolutional layers of the encoder network are attached to one Bi-LSTM recurrent neural network layer to do the classification task. Besides, the performance of both proposed models is further compared to two regular deep learning models having the same layers’ structure, except that the decoder network layers are completely removed. These two models are trained in a supervised manner to only do the classification task. By quantitatively evaluating the performance of the proposed models using different EEG segment lengths, our new approach of using SDCAE will prove to be a very good candidate for producing one of the most accurate seizure detection systems.

## Materials and Methods

### Dataset

**FIGURE 1:**
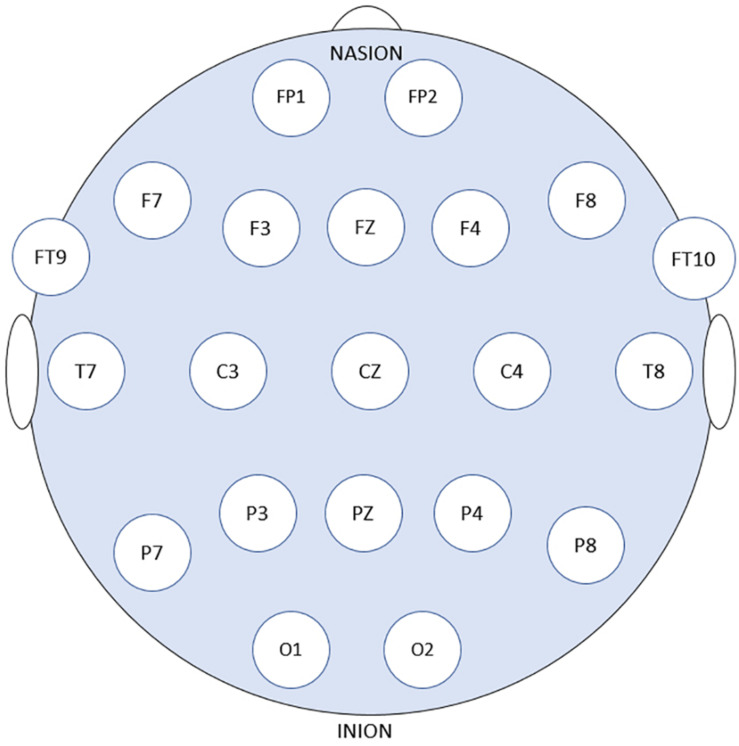
21 EEG electrode positions based on the 10–20 system using modified combinatorial nomenclature.

Patients’ data obtained from the online Children’s Hospital Boston–Massachusetts Institute of Technology (CHB–MIT) Database were used to assess and measure the efficacy of the proposed models. The dataset is recorded at Boston Children’s Hospital and consists of long-term EEG scalp recordings of 23 pediatric patients with intractable seizures (Shoeb, 2009). 23 channels EEG signals recordings are collected using 21 electrodes whose names are specified by the International 10–20 electrode positioning system using the modified combinatorial nomenclature as shown in Figure 1. The signals are then sampled at 256 Hz and the band-pass filtered between 0 and 128 Hz.

In this study, 16 out of the 23 pediatric patients are selected for the assessment of the classification models. More details about the selected patients are listed in Table 1. Seizures less than 10 s are too short so, all Chb16’s seizures were not considered for testing (Gao et al., 2020). The seizures of the two patients (Chb12 and Chb13) were omitted due to the excess variations in channel naming and electrode positioning swapping. Four patients (Chb04, Chb15, Chb18, and Chb19) have been excluded since they are 16 years of age and older because the aim is to research seizure detection in young children.

**TABLE 1:** Seizure information of the selected patients.

Patient#	Gender-age	Number of seizures	Total seizures duration (s)
Chb01	F-11	7	442
Chb02	M-11	2	172
Chb03	F-14	7	402
Chb05	F-7	5	558
Chb06	F-1.5	10	153
Chb07	F-14.5	3	325
Chb08	M-3.5	5	919
Chb09	F-10	4	276
Chb10	M-3	7	447
Chb11	F-12	3	806
Chb14	F-9	8	169
Chb17	F-12	3	293
Chb20	F-6	8	294
Chb21	F-13	4	199
Chb22	F-9	3	204
Chb23	F-6	7	424
Total		86	6083

Typically, epileptic patients have limited numbers of seizures that span much shorter times relative to seizure-free periods. A discrepancy between the number of ictal and interictal EEG data segments is often present. To surmount the bias in the training process of the classification models in which classifiers tend to favor the class with the largest number of segments, and as a design choice, the number of interictal segments is chosen to be equal to the number of ictal segments while forming the final dataset. Downsampling the original interictal dataset can be found in previous work as in Wei et al. (2019), Gao et al. (2020). Non-overlapped EEG segments of 1, 2, and 4 s duration were tested for evaluating the proposed models. A single EEG segment is represented as a matrix whose dimension is (*L* × *N*) where *L* is the sequence length = 256 × segment duration and *N* is the number of channels. As an example, one 2-s segment is represented as a 512 × 23 matrix. The EEG dataset is then formed by putting all the ictal and interictal segments in one matrix whose dimension is (*2KL* × 23) where *K* is the number of the ictal or interictal segments and *L* is as defined before.

### Dataset Preparation

To prepare the EEG dataset before the training phase, all segments combined are pre-processed by applying *z*-score normalization for all channels at one to ensure that all values are standardized by having a zero mean (μ) and unit standard deviation (σ) using the Eq. (1)

(1) $$x=\frac{x-\mu }{\sigma }$$

Next, as a batch, the whole dataset values are scaled to the [0, 1] range using Min–Max normalization to ensure that the original and the reconstructed segments have the same range of values. Finally, the channel’s dimension of the segments is extended by one column to be more suitable for the AE to be used.

### Proposed Systems Architecture

**FIGURE 2:**
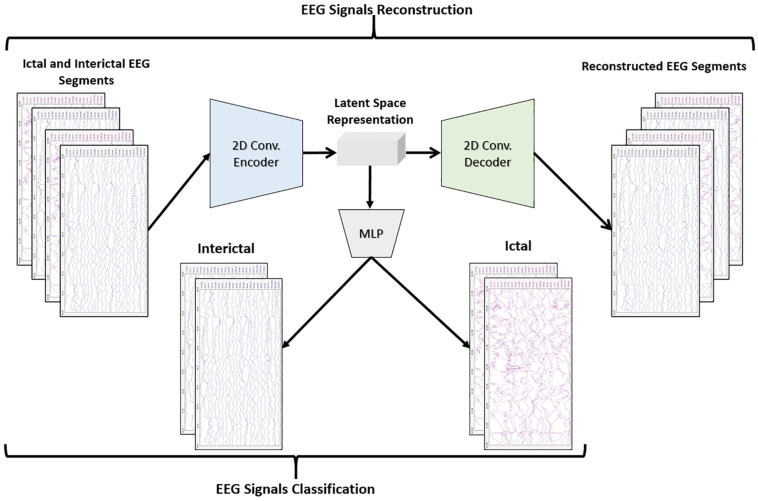
Block diagram of 2D-DCAE + MLP model for seizure detection.

The objective of the article is to build accurate and reliable deep learning models for epileptic seizure detection based on differentiating between two classes of epileptic brain states, interictal and ictal. The proposed models automatically learn powerful features that help to achieve a high classification accuracy of minimally pre-processed EEG signals. Our target is to eliminate the overhead induced by the exhausting manual feature extraction process and also replacing complex systems that require long training times with a much simpler, faster, and more efficient system that benefits from the structure and functionality of AEs. An AE neural network consists of two subnetworks: an encoder and a decoder. The encoder network is used for compressing (encoding) the input information (EEG signals in our case) into a lower-dimensional representation and the decoder is used in a reverse way to decompress or reconstruct the original signal. AE-based compression is accomplished by continually training a network to reconstruct its input while trying to minimize a loss function between the original input and the reconstructed one. 2D-DCAE-based models are proposed for automatically learning inherent signal features from labeled EEG segments while being trained in a supervised way. Figure 2 shows the block diagram of the first proposed model which consists of a 2D-DCAE where the encoder output, the latent space representation, is also fed into an MLP network to perform the classification task.

**FIGURE 3:**
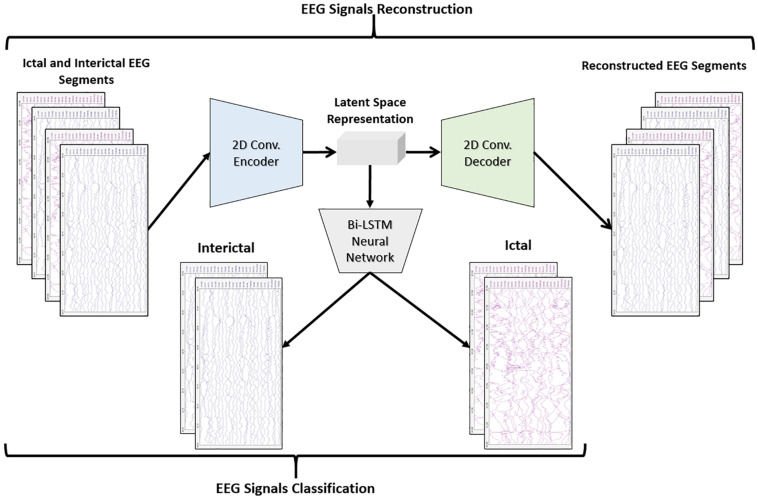
Block diagram of 2D-DCAE + Bi-LSTM model for seizure detection.

Figure 3 shows the block diagram of the second proposed model which consists of a 2D-DCAE but in this case, the encoder output which is the latent space representation is feed into a Bi-LSTM recurrent neural network to perform the classification task.

**FIGURE 4:**
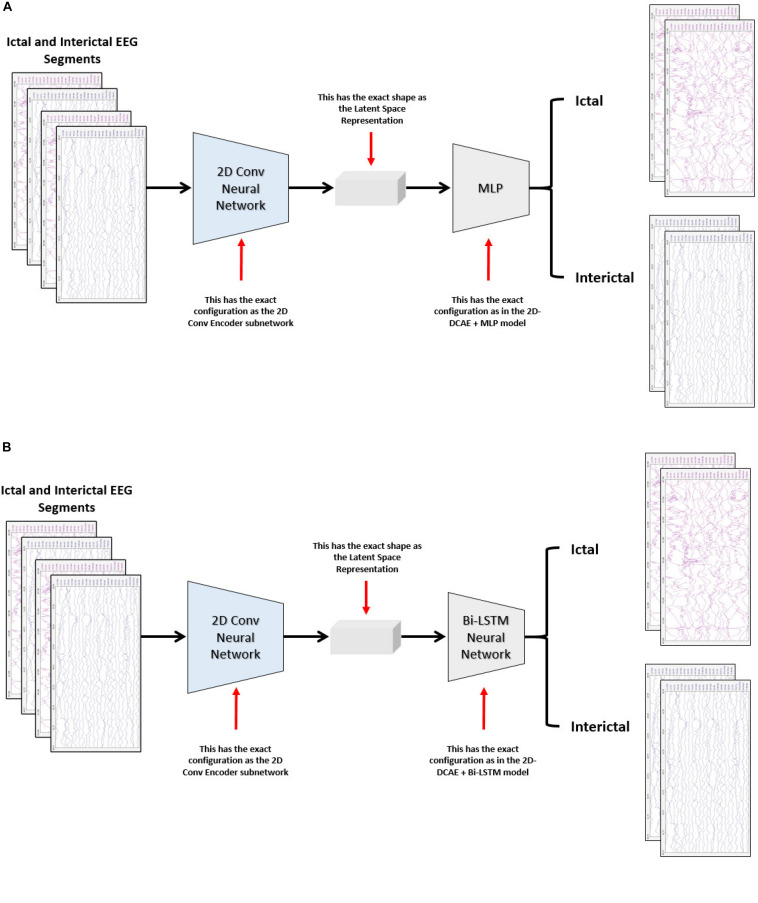
Block diagram of the two-dimensional deep convolutional neural network-based models for seizure detection, **(A)** 2D-DCNN + MLP model and **(B)** 2D-DCNN + Bi-LSTM model.

The performance of the two proposed models will be compared with two other models. One of the new models comprises a two-dimensional deep convolutional neural network (2D-DCNN) connected to an MLP, Figure 4A, while a 2D-DCNN is connected to a Bi-LSTM to form the second model, Figure 4B.

### Two-Dimensional Deep Convolutional Autoencoder

Convolutional neural networks are a special class of feedforward neural networks that are very well-suited for processing multidimensional data like images or multi-channel EEG signals. Applications of CNNs in a variety of disciplines, such as computer vision and pattern recognition, have recorded very impressive outcomes (Krizhevsky et al., 2017). This is due to its great ability to hierarchically learn excellent spatial features for the representation of data of different types. The parameter sharing and sparse connections properties of CNNs make them much more memory-savers compared to MLPs networks that consist of fully connected layers. As a result of these advantages, a two-dimensional convolution autoencoder stacked with convolution and pooling layers is proposed in this work rather than a standard AE that uses only fully connected layers.

The encoder subnetwork of the AE is a CNN consists of four convolutional layers and four max-pooling layers stacked interchangeably. The convolutional layers are responsible for learning the spatial and temporal features in the input EEG signals segments while the max-pooling layers are used for dimensionality reduction by downsampling. A single convolutional layer is made up of filters (kernels) consisting of trainable parameters (weights) that slide over and convolve with the input to generate feature maps where the number of feature maps equals the number of the applied filters. A configurable parameter (stride) controls how much the filter window is sliding over the input. The pooling layer performs down-sampling by lowering the dimension of the feature maps to reduce computational complexity. The low dimensional output of the encoding network is called latent space representation or bottleneck. On the other side, the decoder subnetwork consists of four convolutional layers and four upsampling layers which are also deployed interchangeably and are used to reconstruct the original input.

In all models, in the encoder network, the convolutional layers are configured with 32, 32, 64, and 64 filters, respectively. In the decoder network, the first three convolutional layers are configured with 64, 32, and 32 filters while the last layer has only one filter. All convolutional layers have a kernel size of 3 × 2, and a default stride value equals one. To keep the height and width of the feature maps at the same values, all convolutional layers are configured using the same padding technique. The activation function used in all convolutional layers, except the last layer, is the rectified linear unit (ReLU) defined in Eq. (2) because of its sparsity, computational simplicity, and sturdiness against noise in the input signals (Goodfellow et al., 2017).

(2) f⁢(x)=max⁢{0,x}

where *x* is the weighted sum of the inputs and *f*(*x*) is the ReLU activation function.

The final convolutional layer of the 2D-DCAE uses the sigmoid activation function defined in Eq. (3) to generate an output in the range [0, 1].

(3) $$y=\frac{1}{1+e^{-x}}$$

where *x* is the weighted sum of the inputs and *y* is the output of the activation function.

All max-pooling layers are configured to perform input downsampling by taking the maximum value over windows of sizes (2, 2) except the last layer that uses a window of size (2, 3). The first upsampling layer does its job by interpolating the rows and columns of the input data using a size (2, 3) while the last three upsampling layers use (2, 2) sizes.

Our models apply the Batch Normalization (batch norm) technique for speeding up and stabilizing the training process and to ensure high performance. The batch norm transform (Ioffe and Szegedy, 2015) is defined as:

(4) $$B\,N_{\gamma ,\beta }\,\left(x_{i}\right)=\gamma \,\frac{x_{i}-\mu_{B}}{\sqrt{\sigma_{B}^{2}+\in }}+\beta$$

where an input vector *x*_*i*_ is normalized within a mini-batch *B* = {*x*_1_,*x*_2_…*x*_*m*_} having a mean μ_*B*_and variance $\sigma_{B}^{2}$. β and γ are two parameters that are learned jointly and used to scale and shift the normalized value while ∈ is a constant added for numerical stability. Four batch normalization layers are deployed between the four convolutional and max-pooling layers of the encoder subnetwork.

### Proposed Classification Models

#### Two-Dimensional Deep Convolution Autoencoder + MLP

**FIGURE 5:**
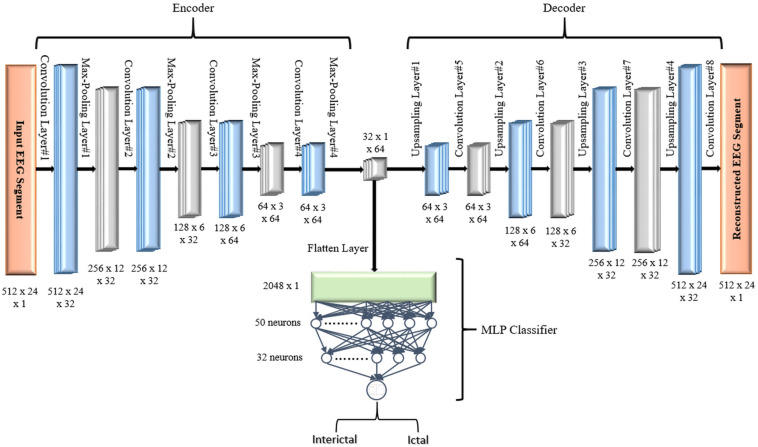
Proposed 2D-DCAE + MLP architecture (assuming that an EEG segment length is 2 s).

In the first proposed model depicted in Figure 5, the output of the decoder subnetwork (the latent space representation) is also converted from its multi-dimensional form to a vector using a flatten layer and then fed into an MLP network-based classifier. The MLP network consists of two hidden fully connected layers having 50 and 32 neurons (units), respectively. Both layers use the Relu activation function. The output layer of the MLP has a sigmoid activation function whose output represents the probability that an input EEG segment belongs to one of the classes.

#### Two-Dimensional Deep Convolution Autoencoder + Bi-LSTM

**FIGURE 6:**
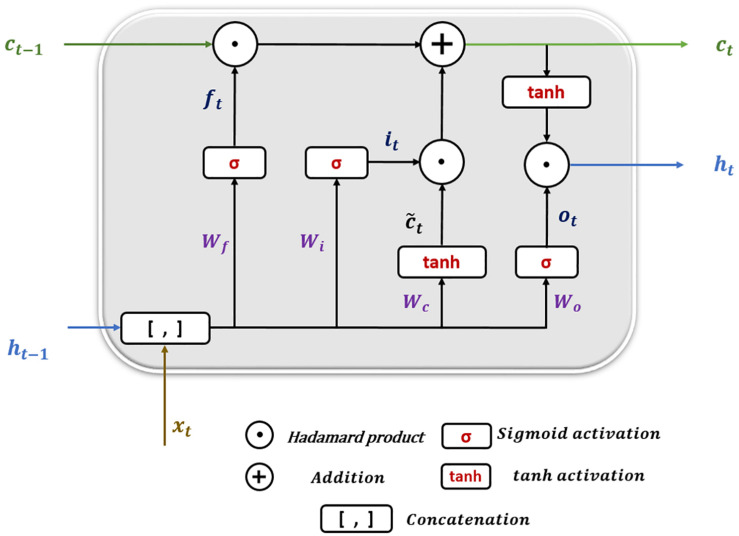
Long short-term memory cell structure.

Long short-term memory (LSTM) is a particular architecture of recurrent neural networks. It was developed to solve numerous problems that vanilla RNNs suffer during training using backpropagation over Time (BPTT) (Mozer, 1989) such as information morphing and exploding and vanishing gradients (Bengio et al., 1994). By proposing the concept of memory cells (units) with three controlling gates, LSTMs are capable of maintaining gradients values calculated by backpropagation during network training while preserving long-term temporal dependencies between inputs (Hochreiter and Schmidhuber, 1997). Figure 6 shows the structure of a single LSTM cell.

The following equations show how information is processed inside the LSTM cell.

(5) $$f_{t}=\sigma \left(W_{f}.\left[h_{t-1},x_{t}\right]+b_{f}\right)$$

(6) $$i_{t}=\sigma \left(W_{i}.\left[h_{t-1},x_{t}\right]+b_{i}\right)$$

(7) $$o_{t}=\sigma \left(W_{o}.\left[h_{t-1},x_{t}\right]+b_{o}\right)$$

(8) $${\tilde{c}}_{t}=t\,a\,n\,h\,\left(W_{c}\cdot \left[h_{t-1},x_{t}\right]+b_{c}\right)$$

(9) $$c_{t}=f_{t}\,⊙c_{t-1}+i_{t}\,⊙{\tilde{c}}_{t}$$

(10) $$h_{t}=o_{t}\,⊙t\,a\,n\,h\,\left(c_{t}\right)$$

where *x_t* is the input at time *t* in a sequence *X* = (*x*_1_,*x*_2_,*x*_3_,.,*x*_*n*_) of *n* time steps. *h*_*t–1*_ and c_t−1_ are the hidden state output and cell state at the previous time step, respectively. h_t_ and *c_t* are the current hidden state and cell state. *f_t*,*i*_*t*_, and *o_t* are the forget, input, and output gates. *W* and *b* represent the weights and biases matrices and vectors while σ is the sigmoid (logistic) function and ⊙ is the Hadamard product operator. The memory cell starts operation by selecting which information to keep or forget from the previous states using the forget gate *f_t*. Then, the cell calculates the candidate state ${\tilde{c}}_{t}$. After that, using the prior cell state c_t−1_ and the input gate *i_t*, the cell decides what further information to write to the current state *c*_*t*_. Finally, the output gate *o_t* calculates how much state information h_t_ will be transported to the next time step. Note that, in Figure 6, the biases and the multiplication operations between the matrix of the concatenated input and the hidden state, and the weight matrices are not shown to make the figure simpler.

**FIGURE 7:**
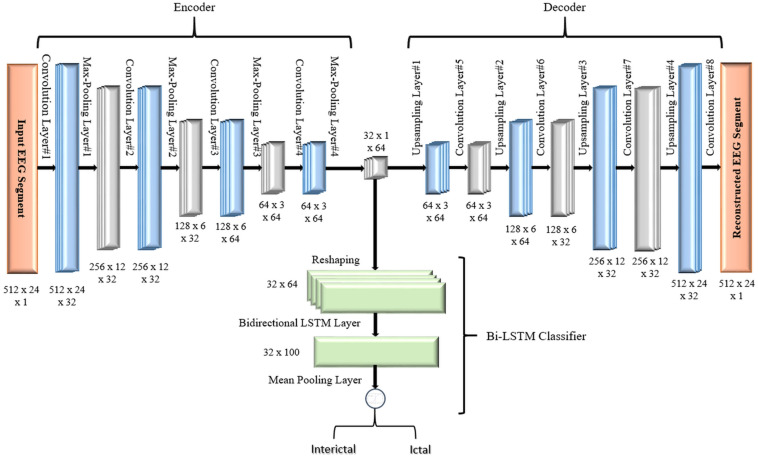
Proposed 2D-DCAE + Bi-LSTM architecture (assuming that an EEG segment length is 2 s).

In the second proposed model, the output of the decoder subnetwork is fed into a Bi-LSTM recurrent neural network-based classifier as shown in Figure 7.

**FIGURE 8:**
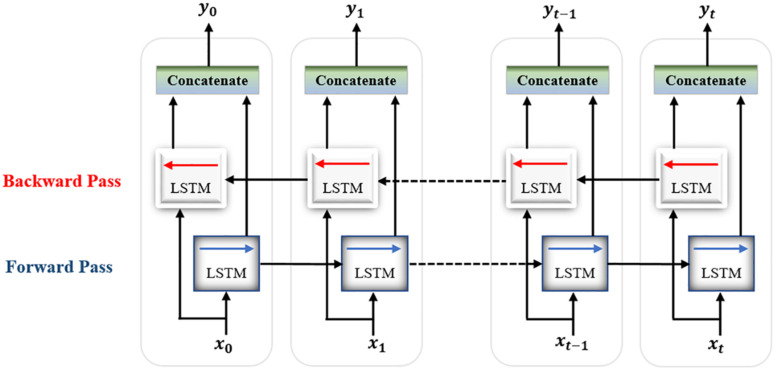
Unrolled single-layer Bi-LSTM network.

For classification, a single-layer Bi-LSTM network consisting of two LSTM blocks (cells) is used in this model. The Bi-LSTM network architecture is similar to the standard unidirectional LSTM architecture, except that both LSTM blocks process the output of the encoder, reshaped as a sequence, simultaneously in two opposite directions instead of one direction. After passing through the entire input sequence, the average of the two outputs of both blocks concatenated together is computed and used for the classification task. Bi-LSTMs are useful in that they take into account the temporal dependence between the current input at a certain time and its previous and subsequent counterparts, which offers a strong advantage for enhancing the classification results (Abdelhameed et al., 2018b). Figure 8 shows a single-layer Bi-LSTM network unrolled over n time steps.

The Bi-LSTM layer is configured to have 50 units and to overcome overfitting, the dropout regularization technique is used with a value of 0.1. As in the first model, the sigmoid activation function is used to predict the EEG segment class label.

#### Loss Functions and Optimizer

As the SDCAE is performing the two tasks of input reconstruction and classification simultaneously, both proposed models are designed to minimize two losses during network training. The first loss is the supervised classification loss (CL) between the predicted and actual class labels. The binary cross-entropy, defined in Eq. (11), is chosen as the loss function.

(11) $$C\,L=-\frac{1}{N}\,\sum_{i=0}^{N-1}y_{i}\cdot \log\,\left({\hat{y}}_{i}\right)+\left(1-y_{i}\right)\cdot \log\,\left(1-{\hat{y}}_{i}\right)$$

where ${\hat{y}}_{i}$ is the predicted model output for a single EEG segment, and *y_i* is the corresponding actual class label in a training batch equals *N.*

The second loss is the loss of reconstruction (RL) between the input EEG segments and their reconstructed equivalents decoded by the DCAE and the mean square error defined in Eq. (12) is utilized for calculating this loss.

(12) $$R\,L=\frac{1}{N}\,\sum_{i=0}^{N-1}\frac{1}{m\,n}\,\sum_{j=0}^{m-1}\sum_{k=0}^{n-1}{\left(y_{j\,k}-{\hat{y}}_{j\,k}\right)}^{2}$$

Where an *y*_*jk*_ is the original value at the position indexed by *j*, *k* in an input EEG segment matrix of size (*m* × *n*), ${\hat{y}}_{j\,k}$ is the reconstructed value and *N* is the number of segments defined as before.

There is no much difference between training a deep learning model with a single output or a deep learning model with multiple outputs. In the latter case as in our proposed SDCAE models, the total loss (TL) of a model is calculated as the weighted summation of the CL and the reconstruction loss (RL) as in Eq. (13)

(13) $$T\,L=w_{c}\times C\,L+w_{r}\times R\,L$$

where *w*_*c*_and *w*_*r*_are the weights and can have any values in the interval (0,1]. In our design, *w*_*c*_is chosen to be 0.5 while *w_r* equals to 1.

The backpropagation of the loss in both subnetworks starts by calculating two partial derivatives (gradients): $\frac{\partial T\,L}{\partial C\,L}$ and $\frac{\partial T\,L}{\partial R\,L}$. All other gradients are then calculated using the chaining rule and the weights and biases are then updated in the same way as typical deep learning models.

Different optimizers such as Stochastic Gradient Descent (SGD; Bottou, 2004), root mean square propagation (RMSProp; Tieleman and Hinton, 2012), ADADELTA (Zeiler, 2012), and Adam (Kingma and Ba, 2014) have been tested while training the SDCAE. Eventually, based on different models’ performances, Adam optimizer was the chosen optimizer with a learning rate set at 0.0001.

### Data Selection and Training

The performance of the two proposed SDCAE seizure detection models (DCAE + MLP), and (DCAE + Bi-LSTM) is evaluated against that of two regular deep learning models (DCNN + MLP), and (DCNN + Bi-LSTM) using EEG segments of three different lengths. That means a total number of twelve models will be tested and assessed using various performance measures.

A stratified 10-fold cross-validation methodology (He and Ma, 2013). is used to prepare the dataset for training and to evaluate the performance of all models to test their strength and reliability while classifying unseen data. In this methodology, the investigated EEG dataset (containing both interictal and ictal data segments) is randomly divided into ten equal subsamples or folds where the balanced distribution of both classes (ictal and interictal) is preserved within each fold. One ten percent of the dataset (a subsample) is marked as the testing set (testing fold) while the remaining nine folds of the dataset collectively are used as the training set. The cross-validation process is repeated for ten iterations, with each of the 10-folds used exactly once as the testing set. Within each iteration, all models are trained for 200 epochs using a batch size of 32. The average and standard deviation of the classification results of the 10 iterations are calculated to produce the final estimations for different evaluation measures.

### Models Performance Evaluation

Various statistical metrics commonly used in the literature such as accuracy, sensitivity (recall), specificity, precision, and F1-score (Sokolova and Lapalme, 2009) have been calculated to assess the classification efficiency of the models against the testing set, in each of the ten iterations of the 10-fold cross-validation. These evaluation metrics are defined as follows:

(14) $$A\,c\,c\,u\,r\,a\,c\,y=\frac{T\,P+T\,N}{T\,P+T\,N+F\,P+F\,N}\times 100\%$$

(15) $$S\,e\,n\,s\,i\,t\,i\,v\,i\,t\,y\,\left(R\,e\,c\,a\,l\,l\right)=\frac{T\,P}{T\,P+F\,N}\times 100\%$$

(16) $$S\,p\,e\,c\,i\,f\,i\,c\,i\,t\,y=\frac{T\,N}{T\,N+F\,P}\times 100\%$$

(17) $$P\,r\,e\,c\,i\,s\,i\,o\,n=\frac{T\,P}{T\,P+F\,P}\times 100\%$$

(18) $$F\,1-s\,c\,o\,r\,e=2\times \frac{P\,r\,e\,c\,i\,s\,i\,o\,n\times R\,e\,c\,a\,l\,l}{P\,r\,e\,c\,i\,s\,i\,o\,n+R\,e\,c\,a\,l\,l}\times 100\%$$

where *P* denotes the number of positive (ictal) EEG segments while *N* denotes the number of negative (interictal) EEG segments). *TP* and *TN* are the numbers of true positives and true negatives while *FP* and *FN* are the numbers of false positives and false negatives, respectively. In this study, accuracy is defined as the percentage of the correctly classified EEG segments belonging to any state (ictal or interictal), sensitivity is the percentage of correctly classified ictal state EEG segments, specificity is the percentage of correctly classified interictal state EEG segments, while precision determines how many of the EEG segments classified as belonging to the ictal state are originally ictal state EEG segments. Finally, the F1-score combines the values of precision and recall in a single metric.

### Models Implementation

The Python programming language along with many supporting libraries and in particular, the Tensorflow machine learning library’s Keras deep learning API, has been used to develop our models. Due to the variations in the hardware resources and different GPU specifications, we have chosen not to include the computational times of training and testing the proposed models as a metric in our comparisons especially since we are developing our models using external resources provided by Google Colaboratory online environment that runs on Google’s cloud servers.

## Results

**FIGURE 9:**
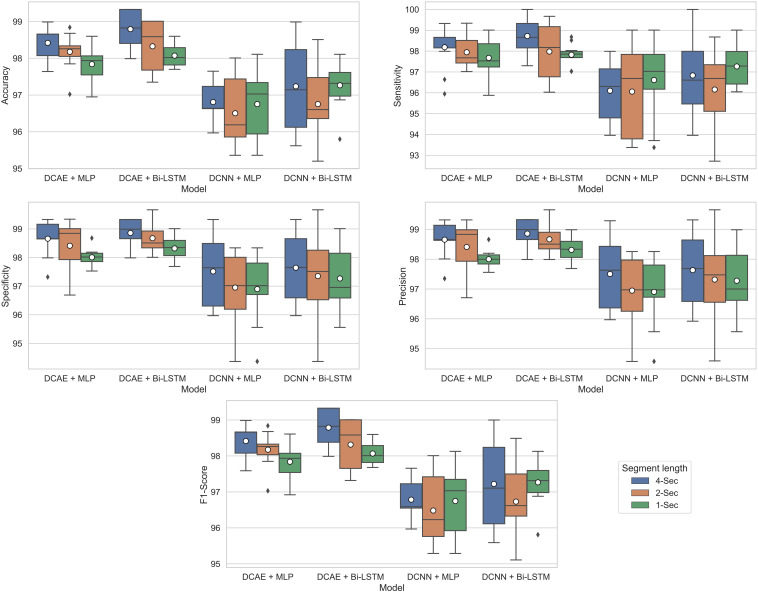
Boxplots showing ranges of performance metrics percentages calculated based on the 10-fold cross-validation results.

For each of the four models, Figure 9 shows the ranges of values of the five performance metrics calculated based on the 10-Fold cross-validation classification results of the EEG segments of lengths 1, 2, and 4 s. The mean and standard deviation of all metrics over the 10-folds are then calculated and summarized in Table 2.

**TABLE 2:** Classification results using different EEG segment lengths.

EEG segment length	Model	Accuracy (%)	Sensitivity (%)	Specificity (%)	Precision (%)	F1-score (%)
1 s	DCAE + MLP	97.84 ± 0.44	97.67 ± 0.88	98.01 ± 0.30	98.00 ± 0.30	97.84 ± 0.46
	DCAE + Bi-LSTM	98.07 ± 0.31	97.83 ± 0.52	98.32 ± 0.43	98.31 ± 0.42	98.07 ± 0.32
	DCNN + MLP	96.75 ± 0.88	96.61 ± 1.79	96.90 ± 1.18	96.91 ± 1.11	96.75 ± 0.90
	DCNN + Bi-LSTM	97.27 ± 0.65	97.27 ± 0.95	97.27 ± 1.09	97.28 ± 1.06	97.27 ± 0.64
2 s	DCAE + MLP	98.18 ± 0.48	97.95 ± 0.71	98.41 ± 0.90	98.41 ± 0.88	98.18 ± 0.48
	DCAE + Bi-LSTM	98.33 ± 0.71	97.98 ± 1.34	98.68 ± 0.50	98.67 ± 0.50	98.32 ± 0.72
	DCNN + MLP	96.51 ± 0.95	96.06 ± 2.12	96.95 ± 1.21	96.95 ± 1.14	96.48 ± 0.98
	DCNN + Bi-LSTM	96.76 ± 1.02	96.16 ± 1.98	97.35 ± 1.54	97.32 ± 1.46	96.73 ± 1.05
4 s	DCAE + MLP	98.42 ± 0.48	98.19 ± 1.02	98.66 ± 0.61	98.65 ± 0.59	98.42 ± 0.50
	DCAE + Bi-LSTM	98.79 ± 0.53	98.72 ± 0.77	98.86 ± 0.53	98.86 ± 0.53	98.79 ± 0.53
	DCNN + MLP	96.81 ± 0.50	96.10 ± 1.43	97.52 ± 1.20	97.50 ± 1.16	96.79 ± 0.51
	DCNN + Bi-LSTM	97.24 ± 1.20	96.84 ± 1.79	97.65 ± 1.28	97.63 ± 1.28	97.22 ± 1.21

**FIGURE 10:**
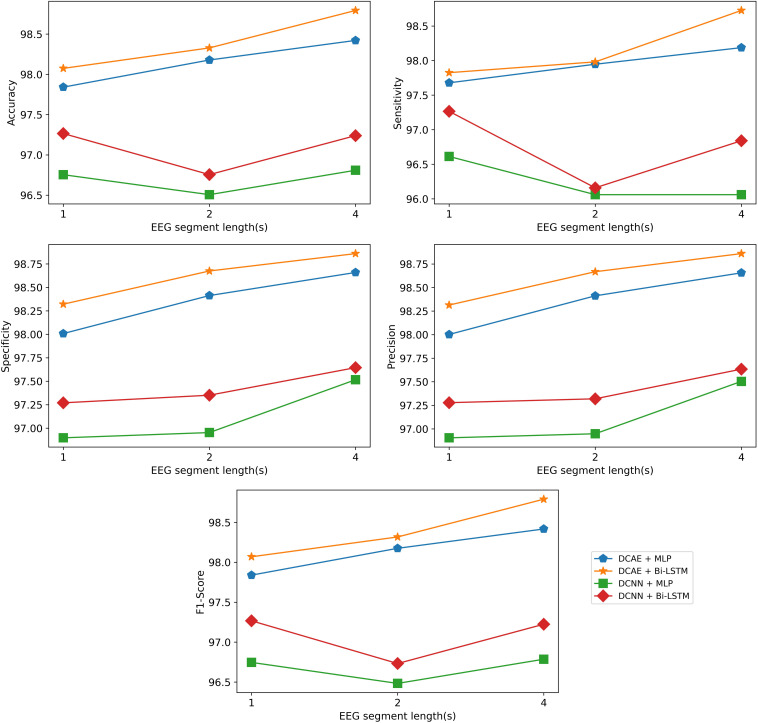
Visualization of the classification results of the models using different EEG segment lengths.

The same results are interpreted visually in Figure 10.

## Discussion

As can be seen from the results, for all EEG segment lengths and evaluation metrics, the two proposed SDCAE models (DCAE + MLP and DCAE + Bi-LSTM) have outperformed the other two models (DCNN + MLP and DCNN + Bi-LSTM) that do not use AEs. Furthermore, as highlighted in Table 2, using a segment length of 4 s, the DCAE + Bi-LSTM model has achieved the highest performance in terms of all evaluation metrics among all other combinations of models. It is also interesting to see that in all SDCAE models, a 4-s EEG segment length is the best choice to get the best classification performance. Generally, it can be noticed that all models that utilized a Bi-LSTM for classification have accomplished better results compared to their counterpart models that use MLP-based classifiers using the same EEG segment lengths. That can be explained as Bi-LSTM networks are more capable to learn better temporal patterns from the generated latent space sequence better than MLP networks. Finally, by comparing the standard deviations in the evaluation metrics values for all models, it is clear that the results of the SDCAE models mostly have less dispersion compared to the other models, which means that the SDCAE models’ performance is more consistent across all cross-validation iterations.

**FIGURE 11:**
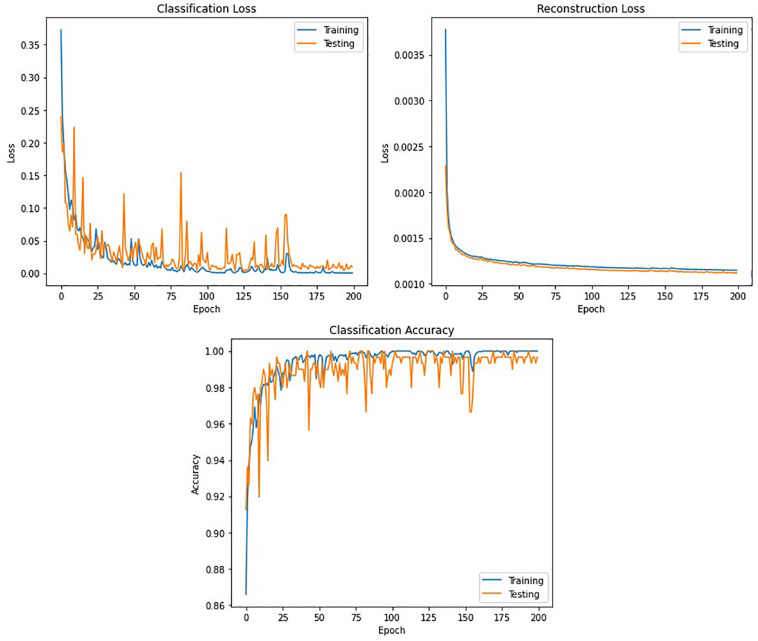
Accuracy and loss curves against the number of epochs obtained while training the DCAE + Bi-LSTM model.

Figure 11 shows the classification accuracy, CL, and RL curves for the training and testing datasets obtained while training the winning model (DCAE + Bi-LSTM) in one of the iterations of the 10-fold cross-validation.

### Statistical Analysis

The non-parametric Kruskal–Wallis *H* test (Kruskal and Wallis, 1952) is used to test the statistical significance of the classification results of the two proposed models (DCAE + MLP and DCAE + Bi-LSTM). For simplicity, the test results for comparing the evaluation metrics of the models obtained using an EEG segment length of 4 s will be demonstrated. When comparing DCAE + Bi-LSTM with the two models (DCNN + MLP and DCNN + Bi-LSTM), the Kruskal–Wallis *H* test produced *p*-value = 0.0005 for accuracy, *p*-value = 0.02 for sensitivity, *p*-value = 0.025 for specificity, *p*-value = 0.005 for precision, and *p*-value = 0.001 for F1-score. Also, when comparing DCAE + MLP with the same two models, the statistical test showed *p*-value = 0.003 for accuracy, *p*-value = 0.011 for sensitivity, *p*-value = 0.083 for specificity, *p*-value = 0.019 for precision, and *p*-value = 0.002 for F1-score. For all performance assessment metrics, nearly all comparisons yielded a *p*-value lower than 0.05, apart from only one *p*-value for specificity. This shows the disparity in the statistical significance between the outcomes of all the proposed models.

### Comparison With Other Methods

In the literature, not all previous work uses the same set of metrics for evaluating the performance of the seizure classification algorithms. So, comparisons based on the most commonly used metrics which are accuracy, sensitivity, and specificity, will only be provided in this section. Table 3 summarizes the comparison between our best performing model and some state-of-the-art methods that use deep neural networks for feature extraction and classification of seizures. In Yuan et al. (2017), various stacked sparse denoising autoencoders (SSDAE) have been tested and compared for feature extraction and classification after preprocessing using short-time Fourier transform (STFT). The best accuracy they obtained was 93.82% using a random selection of training and testing datasets. Ke et al. (2018) combined global maximal information coefficient (MIC) with visual geometry group network (VGGNet) for feature extraction and classification. Using fivefold cross-validation, they achieved 98.1% accuracy, 98.85% sensitivity, and 97.47% specificity. Using fast Fourier transform (FFT) for frequency domain analysis and CNN, the authors (Zhou et al., 2018) performed patient-specific classifications between the ictal and interictal signals. Relying on sixfold cross-validation, the average of the evaluation metrics for all patients was 97.5% accuracy, 96.86% sensitivity, and 98.15% specificity. Finally, in Hossain et al. (2019), the authors used a 2D-CNN model to extract spectral and temporal characteristics of EEG signals and used them for patient-specific classification using a random selection of training and testing datasets. They got 98.05% accuracy, 90% sensitivity, and 91.65% specificity for the cross-patient results. Following the previous comparison, the results obtained by our model have shown to be superior to some of the state-of-the-art systems which all lack the proper statistical analysis for significance testing.

**TABLE 3:** Comparison between our best performing model and previous methods using the same dataset.

Methods	Features extraction	Data selection	Accuracy (%)	Sensitivity (%)	Specificity (%)
Yuan et al., 2017	STFT + SSDAE	Random	93.82	N/A	N/A
Ke et al., 2018	MIC+VGGNet	Fivefold CV	98.1	98.85	97.47
Zhou et al., 2018	FFT+CNN	Sixfold CV	97.5	96.86	98.15
Hossain et al., 2019	2D-CNN	Random	98.05	90	91.65
Proposed Work	(DCAE + Bi-LSTM)	10-Fold CV	98.79	98.72	98.86

## Conclusion

A novel deep-learning approach for the detection of seizures in pediatric patients is proposed. The novel approach uses a 2D-SDCAE for the detection of epileptic seizures based on classifying minimally pre-processed raw multichannel EEG signal recordings. In this approach, an AE is trained in a supervised way to classify between the ictal and interictal brain state EEG signals to exploit its capabilities of performing both automatic feature learning and classification simultaneously with high efficiency. Two SDCAE models that use Bi-LSTM and MLP networks-based classifiers were designed and tested using three EEG data segment lengths. The performance of both proposed models is compared to two regular deep learning models having the same layers’ structure, except that the decoder network layers are completely removed. The twelve models are trained and assessed using a 10-fold cross-validation scheme and based on five evaluation metrics, the best performing model was the SDCAE model that uses a Bi-LSTM and 4 s EEG segments. This model has achieved an average of 98.79% accuracy, 98.72% sensitivity, 98.86% specificity, 98.86% precision, and finally an F1-score of 98.79%. The comparison between this SDCAE model and other state-of-the-art systems using the same dataset has shown that the performance of our proposed model is superior to that of most existing systems.

## Data Availability Statement

Publicly available datasets were analyzed in this study. This data can be found here: https://physionet.org/content/chbmit/1.0.0/.

## Ethics Statement

Ethical review and approval was not required for the study on human participants in accordance with the local legislation and institutional requirements. Written informed consent from the participants’ legal guardian/next of kin was not required to participate in this study in accordance with the national legislation and the institutional requirements.

## Author Contributions

AA conceived the presented idea, conducted the analysis, and produced the figures. MB supervised the findings of this work. Both authors discussed the results and contributed to the final manuscript.

## Conflict of Interest

The authors declare that the research was conducted in the absence of any commercial or financial relationships that could be construed as a potential conflict of interest.

## References

[B1] Abdelhameed A. M. Bayoumi M. (2018). “Semi-supervised deep learning system for epileptic seizures onset prediction,” in *Proceedings of the 2018 17th IEEE International Conference on Machine Learning and Applications (ICMLA)*, Orlando, FL. 10.1109/icmla.2018.00191

[B2] Abdelhameed A. M. Bayoumi M. (2019). Semi-supervised EEG signals classification system for epileptic seizure detection. *IEEE Signal Process. Lett.* 26 1922–1926. 10.1109/lsp.2019.2953870

[B3] Abdelhameed A. M. Daoud H. G. Bayoumi M. (2018a). “Deep convolutional bidirectional LSTM recurrent neural network for epileptic seizure detection,” in *Proceedings of the 2018 16th IEEE International New Circuits and Systems Conference (NEWCAS)*, Montreal, QC. 10.1109/newcas.2018.8585542

[B4] Abdelhameed A. M. Daoud H. G. Bayoumi M. (2018b). “Epileptic seizure detection using deep convolutional autoencoder,” in *Proceedings of the 2018 IEEE International Workshop on Signal Processing Systems (SiPS)*, Cape Town. 10.1109/sips.2018.8598447

[B5] Acharya U. R. Oh S. L. Hagiwara Y. Tan J. H. Adeli H. (2018). Deep convolutional neural network for the automated detection and diagnosis of seizure using EEG signals. *Comput. Biol. Med.* 100 270–278. 10.1016/j.compbiomed.2017.09.017 28974302

[B6] Acharya U. R. Sree S. V. Swapna G. Martis R. J. Suri J. S. (2013). Automated EEG analysis of epilepsy: a review. *Knowled. Based Syst.* 45 147–165. 10.1016/j.knosys.2013.02.014

[B7] Bengio Y. Simard P. Frasconi P. (1994). Learning long-term dependencies with gradient descent is difficult. *IEEE Transact. Neur. Netw.* 5 157–166. 10.1109/72.279181 18267787

[B8] Bottou L. (2004). *Stochastic Learning. Advanced Lectures on Machine Learning, LNAI*, Vol. 3176. Berlin: Springer, 146–168.

[B9] Chen G. Xie W. Bui T. D. Krzyżak A. (2017). Automatic epileptic seizure detection in EEG using nonsubsampled wavelet–fourier features. *J. Med. Biol. Eng.* 37 123–131. 10.1007/s40846-016-0214-0

[B10] Epilepsy in Children (2020). *Diagnosis & Treatment HealthyChildren.org.* Available online at: https://www.healthychildren.org/English/health-issues/conditions/seizures/Pages/Epilepsy-in-Children-Diagnosis-and-Treatment.aspx (accessed December 15, 2020).

[B11] Gao Y. Gao B. Chen Q. Liu J. Zhang Y. (2020). Deep convolutional neural network-based epileptic electroencephalogram (EEG) signal classification. *Front. Neurol.* 11:375. 10.3389/fneur.2020.00375 32528398 PMC7257380

[B12] Gogna A. Majumdar A. Ward R. (2017). Semi-supervised stacked label consistent autoencoder for reconstruction and analysis of biomedical signals. *IEEE Transact. Biomed. Eng.* 64 2196–2205. 10.1109/tbme.2016.2631620 27893378

[B13] Goodfellow I. Bengio Y. Courville A. (2017). *Deep Learning.* Cambridge, MA: The MIT Press.

[B14] He H. Ma Y. (2013). *Imbalanced Learning Foundations, Algorithms, and Applications.* Hoboken, NJ: IEEE Press.

[B15] Hochreiter S. Schmidhuber J. (1997). Long short-term memory. *Neur. Comput.* 9 1735–1780. 10.1162/neco.1997.9.8.1735 9377276

[B16] Hossain M. S. Amin S. U. Alsulaiman M. Muhammad G. (2019). Applying deep learning for epilepsy seizure detection and brain mapping visualization. *ACM Transact. Multimed. Comput. Commun. Appl.* 15 1–17. 10.1145/3241056

[B17] Hu W. Cao J. Lai X. Liu J. (2019). Mean amplitude spectrum based epileptic state classification for seizure prediction using convolutional neural networks. *J. Ambient Intell. Human. Comput.* 10.1007/s12652-019-01220-6

[B18] Ioffe S. Szegedy C. (2015). Batch normalization: accelerating deep network training by reducing internal covariate shift. *arXiv* [Preprint]. arXiv:1502.03167.

[B19] Jaiswal A. K. Banka H. (2017). Local pattern transformation based feature extraction techniques for classification of epileptic EEG signals. *Biomed. Signal Process. Control* 34 81–92. 10.1016/j.bspc.2017.01.005

[B20] Ke H. Chen D. Li X. Tang Y. Shah T. Ranjan R. (2018). Towards brain big data classification: epileptic EEG identification with a lightweight VGGNet on global MIC. *IEEE Access* 6 14722–14733. 10.1109/access.2018.2810882

[B21] Kingma D. Ba J. (2014). Adam: a method for stochastic optimization. *ArXiv* [Preprint]. arXiv:1412.6980.

[B22] Krizhevsky A. Sutskever I. Hinton G. E. (2017). ImageNet classification with deep convolutional neural networks. *Commun. ACM* 60 84–90. 10.1145/3065386

[B23] Kruskal W. H. Wallis W. A. (1952). Use of ranks in one-criterion variance analysis. *J. Am. Statist. Associat.* 47 583–621. 10.1080/01621459.1952.10483441

[B24] Mozer M. C. (1989). A focused backpropagation algorithm for temporal pattern recognition. *Comp. Syst.* 3 349–381.

[B25] Orhan U. Hekim M. Ozer M. (2011). EEG signals classification using the K-means clustering and a multilayer perceptron neural network model. *Exp. Syst. Appl.* 38 13475–13481. 10.1016/j.eswa.2011.04.149

[B26] Raghu S. Sriraam N. Hegde A. S. Kubben P. L. (2019). A novel approach for classification of epileptic seizures using matrix determinant. *Exp. Syst. Appl.* 127 323–341. 10.1016/j.eswa.2019.03.021

[B27] Samiee K. Kovacs P. Gabbouj M. (2015). Epileptic seizure classification of EEG time-series using rational discrete short-time fourier transform. *IEEE Transact. Biomed. Eng.* 62 541–552. 10.1109/tbme.2014.2360101 25265603

[B28] Schomer D. L. Lopez da Silva H. F. (2018). *Niedermeyer’s Electroencephalography: Basic Principles, Clinical Applications, and Related Fields.* New York, NY: Oxford University Press.

[B29] She Q. Hu Bo Luo Z. Nguyen T. Zhang L. (2018). A hierarchical semi-supervised extreme learning machine method for EEG recognition. *Med. Biol. Eng. Comput.* 57 147–157. 10.1007/s11517-018-1875-3 30054779

[B30] Shoeb A. H. (2009). *Application of Machine Learning to Epileptic Seizure Onset Detection and Treatment.* Ph.D. thesis, Massachusetts Institute of Technology, Cambridge, MA.

[B31] Sokolova M. Lapalme G. (2009). A systematic analysis of performance measures for classification tasks. *Inform. Process. Manag.* 45 427–437. 10.1016/j.ipm.2009.03.002

[B32] Song Y. Crowcroft J. Zhang J. (2012). Automatic epileptic seizure detection in EEGs based on optimized sample entropy and extreme learning machine. *J. Neurosci. Methods* 210 132–146. 10.1016/j.jneumeth.2012.07.003 22824535

[B33] Tieleman T. Hinton G. (2012). Lecture 6.5-RMSprop: divide the gradient by a running average of its recent magnitude. *COURSERA Neur. Netw. Mach. Learn.* 4 26–31.

[B34] Wang D. Ren D. Li K. Feng Y. Ma D. Yan X. (2018). Epileptic seizure detection in long-term EEG recordings by using wavelet-based directed transfer function. *IEEE Transact. Biomed. Eng.* 65 2591–2599. 10.1109/tbme.2018.2809798 29993489

[B35] Wang X. Zhao Y. Pourpanah F. (2020). Recent advances in deep learning. *Int. J. Mach. Learn. Cybern.* 11 747–750. 10.1007/s13042-020-01096-5

[B36] Wei X. Zhou L. Zhang Z. Chen Z. Zhou Y. (2019). Early prediction of epileptic seizures using a long-term recurrent convolutional network. *J. Neurosci. Methods* 327:108395. 10.1016/j.jneumeth.2019.108395 31408651

[B37] World Health Organization (2020). *Epilepsy.* Available online at: https://www.who.int/en/news-room/fact-sheets/detail/epilepsy (accessed December 5, 2020).

[B38] Yavuz E. Kasapbaşı M. C. Eyüpoğlu C. Yazıcı R. (2018). An epileptic seizure detection system based on cepstral analysis and generalized regression neural network. *Biocybern. Biomed. Eng.* 38 201–216. 10.1016/j.bbe.2018.01.002

[B39] Yuan Y. Xun G. Jia K. Zhang A. (2017). “A multi-view deep learning method for epileptic seizure detection using short-time fourier transform,” in *Proceedings of the 8th ACM International Conference on Bioinformatics, Computational Biology, and Health Informatics*, New York, NY. 10.1145/3107411.3107419

[B40] Zeiler M. D. (2012). ADADELTA: an adaptive learning rate method. *arXiv* [Preprint]. arXiv:1212.5701.

[B41] Zhou M. Tian C. Cao R. Wang B. Niu Y. Hu T. (2018). Epileptic Seizure detection based on EEG signals and CNN. *Fronti. Neuroinform.* 12:95. 10.3389/fninf.2018.00095 30618700 PMC6295451

